# Exogenous TiO_2_ Nanoparticles Alleviate Cd Toxicity by Reducing Cd Uptake and Regulating Plant Physiological Activity and Antioxidant Defense Systems in Rice (*Oryza sativa* L.)

**DOI:** 10.3390/metabo13060765

**Published:** 2023-06-19

**Authors:** Anas Iqbal, Zhaowen Mo, Sheng-Gang Pan, Jian-Ying Qi, Tian Hua, Muhammad Imran, Meiyang Duan, Qichang Gu, Xiang-Bin Yao, Xiangru Tang

**Affiliations:** 1 State Key Laboratory for Conservation and Utilization of Subtropical Agro-Bioresources, College of Agriculture, South China Agricultural University, Guangzhou 510642, China; anasiqbal@scau.edu.cn (A.I.); tianhua@scau.edu.cn (T.H.); meiyang@scau.edu.cn (M.D.);; 2 Scientific Observing and Experimental Station of Crop Cultivation in South China, Ministry of Agriculture and Rural Affairs, Guangzhou 510642, China; 3 Guangzhou Key Laboratory for Science and Technology of Fragrant Rice, Guangzhou 510642, China

**Keywords:** cadmium, heavy metals, titanium dioxide, fragrant rice, antioxidant enzymes, leaf photosynthetic traits, proline content

## Abstract

Cadmium (Cd) is a potentially hazardous element with significant biological toxicity, negatively affecting plant growth and physio-biochemical metabolism. Thus, it is necessary to examine practical and eco-friendly approaches to reduce Cd toxicity. Titanium dioxide nanoparticles (TiO_2_-NPs) are growth regulators that help in nutrient uptake and improve plant defense systems against abiotic and biological stress. A pot experiment was performed in the late rice-growing season (July—November) 2022 to explore the role of TiO_2_-NPs in relieving Cd toxicity on leaf physiological activity, biochemical attributes, and plant antioxidant defense systems of two different fragrant rice cultivars, i.e., Xiangyaxiangzhan (XGZ) and Meixiangzhan-2 (MXZ-2). Both cultivars were cultivated under normal and Cd-stress conditions. Different doses of TiO_2_-NPs with and without Cd-stress conditions were studied. The treatment combinations were: Cd−, 0 mg/kg CdCl_2_·2.5 H_2_O; Cd+, 50 mg/kg CdCl_2_·2.5 H_2_O; Cd + NP1, 50 mg/kg Cd + 50 TiO_2_-NPs mg/L; Cd + NP2, 50 mg/kg Cd + 100 TiO_2_-NPs mg/L; Cd + NP3, 50 mg/kg Cd + 200 TiO_2_-NPs mg/L; Cd + NP4, 50 mg/kg Cd + 400 TiO_2_-NPs mg/L. Our results showed that the Cd stress significantly (*p* < 0.05) decreased leaf photosynthetic efficiency, stomatal traits, antioxidant enzyme activities, and the expression of their encoding genes and protein content. Moreover, Cd toxicity destabilized plant metabolism owing to greater accretion of hydrogen peroxide (H_2_O_2_) and malondialdehyde (MDA) levels at vegetative and reproductive stages. However, TiO_2_-NPs application improved leaf photosynthetic efficacy, stomatal traits, and protein and antioxidant enzyme activities under Cd toxicity. Application of TiO_2_-NPs decreased the uptake and accumulation of Cd in plants and levels of H_2_O_2_ and MDA, thereby helping to relieve Cd-induced peroxidation damage of leaf membrane lipids by enhancing the activities of different enzymes like ascorbate peroxidase (APX), catalase (CAT), peroxidase (POS), and superoxide dismutase (SOD). Average increases in SOD, APX, CAT, and POS activities of 120.5 and 110.4%, 116.2 and 123.4%, 41.4 and 43.8%, and 36.6 and 34.2% in MXZ-2 and XGZ, respectively, were noted in Cd + NP3 treatment across the growth stages as compared with Cd-stressed plants without NPs. Moreover, the correlation analysis revealed that the leaf net photosynthetic rate is strongly associated with leaf proline and soluble protein content, suggesting that a higher net photosynthetic rate results in higher leaf proline and soluble protein content. Of the treatments, the Cd + NP3 (50 mg/kg Cd + 200 mg/L TiO_2_-NPs) performed the best for both fragrant rice cultivars under Cd toxicity. Our results showed that TiO_2_-NPs strengthened rice metabolism through an enhanced antioxidant defense system across the growth stages, thereby improving plant physiological activity and biochemical characteristics under Cd toxicity.

## 1. Introduction

Soil and water contamination by heavy metals is a severe problem across the globe, as they have harmful effects on plants and humans and are transferable from plants to humans via the food chain [1]. Chemical, petroleum-refining, mining, and manufacturing industries account for heavy metal production; in many countries, heavy metals have been declared significant risks for humans and the environment [2]. Phosphate (P) rock contains varying amounts of innate cadmium (Cd), and some of that Cd is transferred to fertilizer products during the manufacturing process [3]. Cd is the most toxic heavy metal present in contaminated soil, posing threats to plants as well as humans [4], as it is highly soluble in water, relatively mobile, and has long half-lives in living organisms [5]. Heavy metals mostly lack biodegradability, so they quickly accumulate in the environment and enter the food chain. In modern agriculture, arable soils are polluted due to the heavy use of chemical fertilizers, particularly P fertilizer [6,7,8]. Several studies point to the impact of mineral P fertilizers as a significant source of Cd contamination in agricultural soils [9]. In Europe, mineral P fertilizers contribute 45% of the total Cd contamination of cropland [10]. With the advancement in industrial applications, emissions, and the worldwide use of phosphate fertilizers, Cd has emerged as a major soil pollutant [11].

Rice (*Oryza sativa* L.) is the main staple food consumed by half of the world’s population and about 60% of China’s population [12,13]. Rice is known as “the grain of life” and is identified as the food of Asian people. Over two billion people in Asia obtain 80% of their energy from rice. Rice has a high nutritional value, including carbohydrates (80%), protein (7–9%), fat (3%), and fiber (3%) [14]. Rice is a major food and an important part of social ceremonies such as festivals and rituals in almost all Asian countries, and the medical system in the region has clearly documented its medical significance. China is a major producer and consumer of rice and ranks first in the world [12]. 

The toxicity caused by Cd has become a dilemma in arable soils worldwide [15]. As Cd is not essential for living organisms, it harms plants and animals even at low concentrations. In rice fields, the rice plants absorb Cd from the contaminated soil through roots, from where it then reaches the plants’ upper parts and ultimately affects the plants’ morphological, physiological, and biochemical activities at different developmental stages [16,17]. Under Cd-stress conditions, the infected plants develop specific morphological abnormalities such as stunted growth, reduction in the concentration of leaf photosynthetic pigments, leaf chlorosis, and plant death [17,18]. Previously, Cd stress has been reported to affect plant protein synthesis, inhibit different enzymatic activities, and disrupt nutrient uptake and transport to other parts of the plants [16,19,20,21]. Therefore, Cd-contaminated soil severely threatens sustainable agriculture and food safety. Moreover, the adverse effects of Cd on human health were first observed in subsistence rice farmers in Japan in the mid-1950s. They contracted Cd poisoning (itai-itai disease) after decades of consuming home-grown rice irrigated with Cd- and Zn-enriched mine wastes [22]. The main effect of Cd on human health is kidney disease; although other adverse effects have been reported (e.g., pulmonary, cardiovascular, and musculoskeletal systems), controversy exists regarding their effects [23,24]. Thus, there is a dire need to investigate and examine practical and eco-friendly approaches to reduce Cd uptake and accumulation in rice plants.

The recent advancement in nano-technological approaches promotes essential and widespread applications of nanoparticles (NPs). Among the NPs, TiO_2_ has been widely used as a good nano fertilizer or nano pesticide to enhance crop yield, reduce crop diseases, and decrease hazardous organic solvents in agrichemicals [25]. Using TiO_2_-NPs in different concentrations, sizes, and exposure subsequently affected the plant biomass [26]. Previously, a positive correlation was reported between the use of TiO_2_-NPs with the availability of nutrients and the growth pattern of plants [27]. TiO_2_-NP application increased shoot length; increased phosphorus accumulation in grains, roots, and shoots in rice; and increased spinach biomass [28]. Applying TiO_2_-NPs in wheat resulted in an increased chlorophyll content [29]. Furthermore, the application of TiO_2_-NPs has been reported to alleviate Cd toxicity and increase plant biomass [27]. Applying nano-TiO_2_ promotes the growth of spinach plants by protecting the sheath assembly of chloroplast from the adverse effects of reactive oxygen species, thereby increasing the effects of antioxidant enzyme functions [30]. In recent studies, application of TiO_2_-NPs enhanced plant biomass by reducing Cd phytotoxicity [27,31]. The efficiency of TiO_2_-NPs depends on their chemical composition, size, surface covering, reactivity, and, most important, the dose at which they are effective [32]. Further, the efficiency of TiO_2_-NPs also depends on the plant species [33]. However, there is a lack of information regarding TiO_2_-NP application to different fragrant rice cultivars and its effects on the physiological and biochemical processes of rice.

Most people prefer fragrant rice because of its optimal flavor and other features [34]. The current research used two fragrant rice varieties, i.e., Meixiangzhan-2 (MXZ-2) and Xiangyaxiangzhan (XGZ), widely cultivated in southern China [35]. As a semi-aquatic tropical crop sown in marshy soils, rice is heavily exposed to the accumulation of certain trace metals such as Cd [5]. Thus, the main objective of the current study was to dissect the functional role of TiO_2_-NPs in alleviating Cd toxicity, thereby enhancing the growth of rice by regulating different physiological and biochemical processes. The mainly focus of the present study was (1) to examine the plant growth, biomass accumulation, and photosynthetic pigment content during different growth stages under Cd-stress conditions, (2) to explore the effect of TiO_2_ on plant whole antioxidant systems under Cd toxicity, and (3) to contribute toward eliminating the potential threats posed by heavy metals to human health and ensuring food security.

## 2. Materials and Methods

### 2.1. Experimental Site and Soil Properties

The pot experiment was performed during the late rice-growing season (July–November) 2022 at South China Agriculture University (SCAU) Research Station (23°15′ N, 113°21′ E) in southern China. The soil at the study site (0–20 cm) exhibits a slightly acidic nature (pH of 5.87). Moreover, the soil comprises 20.78 g kg^−1^ organic matter, 1.16 g kg^−1^ total nitrogen (TN), 90.45 mg kg^−1^ available nitrogen (AN), and 0.96 g kg^−1^ total phosphorus (TP). Details of the soil’s chemical properties are presented in Supplementary Table S1.

### 2.2. Crop Husbandry, Growing Conditions, and Treatment Details

Two different cultivars of rice (*Oryza sativa* L.), MXZ-2 and XGZ, were used for experiments in the current study. These cultivars were collected from the College of Agriculture, South China Agriculture University, Guangzhou, China. The above cultivars were selected because of the same growth pattern and different responses to Cd toxicity. Because of the higher Cd accumulation and distribution to various plant sections under the same Cd stress, our recent research showed that the MXZ-2 fragrant rice cultivar was less Cd-tolerant than the XGZ fragrant rice cultivar [35].

The pot experiment was carried out in the late season (July–November). A complete block design with five replications and six different treatments was used in the experiment. The soil was gathered from the uncontaminated paddy field to a depth of 20 cm, dried, ground, and then poured into plastic pots measuring 30 cm in height by 25 cm in diameter. To reduce experimental error, we ensured that the soil in each experimental pot had the same weight (12 kg per pot). Different concentrations of TiO_2_-NPs and Cd were applied. The treatment combinations were: Cd−, 0 mg/kg CdCl_2_·2.5 H_2_O; Cd+, 50 mg/kg CdCl_2_·2.5 H_2_O; Cd + NP1, 50 mg/kg Cd + 50 TiO_2_-NPs mg/L; Cd + NP2, 50 mg/kg Cd + 100 TiO_2_-NPs mg/L; Cd + NP3, 50 mg/kg Cd + 200 TiO_2_-NPs mg/L; Cd + NP4, 50 mg/kg Cd + 400 TiO_2_-NPs mg/L). In the pots, 15 days before seedling transplantation, full concentrations of Cd and TiO_2_-NPs were thoroughly mixed.

The plastic trays were used for the seed growth, and after 24 days, uniform-size seedlings were selected and transplanted into plastic pots. Each pot contained four hills and three transplanted seedlings of similar size per hill. The recommended dose of nitrogen (N), phosphorus (P), and potassium (K) fertilizer at a rate of 300:150:300 (kg ha^−1^) was applied. We applied 1.80 g of N as urea, 0.90 g P_2_O_2_ as superphosphate, and 2.20 g KCl in the form of potassium chloride. N and KCl were applied in three splits, 60%, 20%, and 20%, as basal, tillering, and panicle-initiation doses. During seedling transplantation, all P_2_O_2_ (100%) was applied as a basal dose two days prior. Uniform flooding irrigation was maintained from planting seedlings to physiological maturity to establish anaerobic conditions in the pots. Usual farming practices, such as insecticide and pesticide application, were applied in all treatments.

### 2.3. Sampling and Analysis

Fresh plant leaves were collected from the two cultivars at the vegetative and reproductive stages. The leaves were preserved at −80 °C to measure photosynthetic pigments and conduct leaf physio-biochemical analysis. Moreover, rice plants were harvested for biomass and Cd accumulation at tillering and heading stages.

#### 2.3.1. Measurement of Leaf Gas Exchange Attributes

The different gaseous exchange aspects, such as net photosynthetic rate (*Pn*), transpiration rate (Tr), stomatal conductance (g_s_), and intercellular CO_2_ concentration (Ci), were measured at tillering and heading stages. A portable photosynthesis system (Li-6800, Li-COR Inc., Lincoln, NE, USA) was used to determine photosynthesis. Fully expanded leaves from each pot during full sunlight (from 9:30 a.m. to 12:30 p.m.) were used for this purpose.

#### 2.3.2. Leaf Scanning Electron Microscopy (SEM) Analysis

A uniform portion (1 mm^2^) from the middle parts of leaves was taken in three replicates for each selected treatment. Distilled water was used to wash the samples before the electron microscopy analysis. A solution of 4% glutaraldehyde and 0.2 M sodium phosphate buffer (pH 6.8) was used to fix the collected samples (6 h, 4 °C). The samples were then washed four times with 0.1 M sodium phosphate buffer (pH 6.8). After this, samples were washed with diluted ethanol; the samples were rinsed twice with isoamyl acetate and freeze-dried. The leaf fragments were fixed firmly on stubs with double-sided tape, and samples were sputter-coated using gold [36]. Finally, a JEOLJSM-6390 LV Scanning Electron Microscope was used to analyze the samples.

#### 2.3.3. Determination of Antioxidant Enzyme Activities

Activities of antioxidant enzymes, including superoxide dismutase (SOD; EC 1.15.1.1), peroxidase (POD; EC1.11.1.6), catalase (CAT; EC 1.11.1.6), and ascorbate peroxidase (APX; EC 1.11.1.6), were determined by using previously described methods [37]. Briefly, fresh rice leaves were homogenized using sodium phosphate buffer (50 mM, pH 7.5). The homogenized sample was centrifuged at 12,000 rpm for 10 min at 4 °C. The supernatant was then collected and used for subsequent assays. In the enzyme extract, SOD activity was measured using an enzyme solution containing methionine (750 mM), NBT (5.2 μM), EDTA (0.1 mM), and PBS (50 mM). The enzymatic activities of POD, CAT, and SOD were measured as previously reported [38]. Moreover, the activity of GR was calculated by the procedure described by Jiang and Zhang [38].

#### 2.3.4. Total RNA Extraction and qRT-PCR Analysis

Total RNA was extracted from the frozen samples using TRIzol reagent (Invitrogen, Carlsbad, CA, USA). Subsequently, according to the previously reported method, qRT-PCR was conducted according to the method of Pfaffl [39]. The RNA was dissolved in DEPC.H_2_O, and further analyses were conducted with a spectrophotometer (Nano-Drop UV-USA 2000, Thermo Fisher Scientific, Waltham, MA, USA). The RNA was reverse-transcribed to cDNA using M-MLVRTase enzyme (Promega, Madison, WI, USA), Oligo primers (dT18) (Promega, Madison, WI, USA), and dNTPs through IQ5 Real-Time PCR (Bio-Rad, Hercules, CA, USA). For subsequent analysis, gene-specific primers, synthesized cDNA template, and SYBR Green mix (Bio-Rad, Hercules, CA, USA) were mixed in a 96-well plate. PCR was performed for the reaction mixture using the following steps: denaturation for 30 s at 95 °C, 40 cycles of annealing at different temperatures for 20 s, followed by a 30 s extension at 72 °C. For relative quantification, rice ACTIN (Os03g50885) (F) 5′-TGCCAAGGCTGAGTACGACGA-3′ and (R) 5′-CAAGCAGGAGGACGGCGATA-3′ were used as reference genes.

The information associated with nucleotide sequences and specific annealing temperatures is presented in Table S2. Three biological repeats were used, and the expression levels were determined by standardizing the Ct value for each gene relative to the ACTIN value; the *2*^−∆∆Ct^ technique was used for quantification as recommended in a previous study [40].

#### 2.3.5. Determination of Malondialdehyde and Hydrogen Peroxide

MDA content was quantified as previously described [41]. H_2_O_2_ was measured from fresh fragrant rice leaf samples using the previously reported method [42].

#### 2.3.6. Measurement of Proline and Protein Content

As previously reported, the proline contents in fresh leaves during vegetative and reproductive stages were quantified [43]. The reaction mixture was extracted with 5 mL toluene, and the red chromophore absorbance in the toluene fraction was checked at 520 nm wavelength. Aliquots of 0.1 g of fresh leaves were homogenized in 50 mM sodium phosphate buffer (1 mM EDTA-Na2, 2% polyvinyl pyrrolidine-40), and the reaction was centrifuged at 10,000× *g* (15 min at 4 °C). The reaction mixture was passed at 595 nm in triplicate, and the final protein contents are reported as mg g^−1^.

#### 2.3.7. Determination of Plant Cd Concentration

The oven-dried samples were finely crushed into powder and then digested using HNO_3_ and HClO_4_ at a ratio of 4:1 (*v*/*v*), and the dilutions were prepared up to 25 mL. The Cd concentrations in roots, leaves, and grains were then measured using a flame atomic absorption spectrometer (AAA 6300 C, Shimadzu, Japan) as previously reported [44].

#### 2.3.8. Statistical Analysis

Relevant ANOVA techniques for completely randomized design were used to analyze the data collected from rice cultivars on biochemical and photosynthetic traits and antioxidant enzyme transcript levels using Statistix 8.1 software (Analytical Software, Tallahassee, FL, USA). Before analysis, data were processed using an arcsine function to normalize the variables. Multiple means for the variables where the effects of experimental factors were significant were compared using Tukey’s post-hoc test. For correlation analysis, linear regression was performed to evaluate the relationship between leaf photosynthetic activity and biochemical traits.

## 3. Results

### 3.1. Effect of TiO_2_-NPs on Leaf Net Photosynthetic Efficiency under Cd Stress

**Figure 1:**
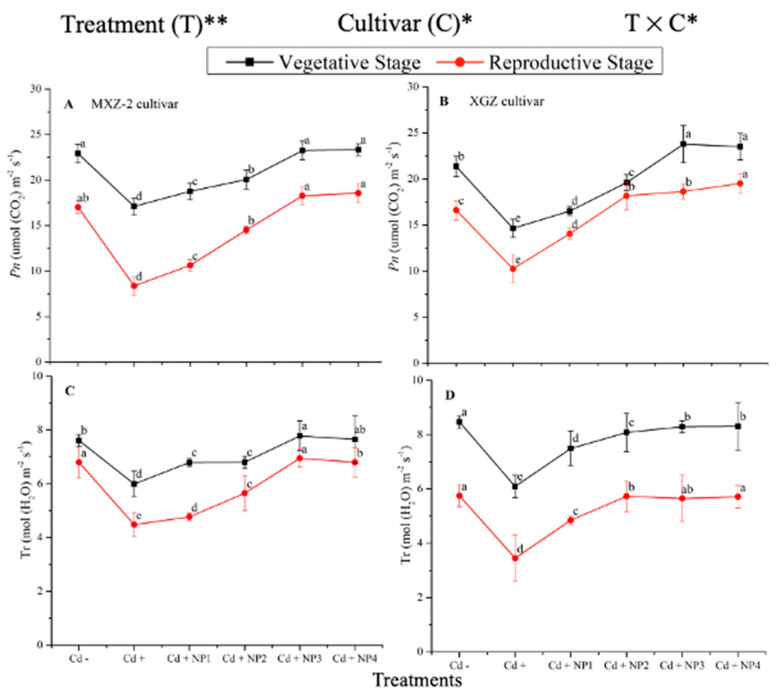
Effect of TiO_2_-NP application and Cd stress on photosynthetic rate (**A**,**B**) and transpiration rate (**C**,**D**) in the leaves of fragrant rice cultivars Meixiangzhan-2 (MXZ-2) and Xiangyaxiangzhan (XGZ) at the vegetative and reproductive stages. Tukey tests were used to compare means for the regimes in both stages, and a simple test based on the Tukey HSD test at 0.05 was used to interpret the results. Error bars are standard errors of the mean. Different letters, such as a, b, c, d & e above the lines, indicate statistical significance at *p* < 0.05. *, ** = significant at 5% and 1%, respectively; Cd−, 0 mg/kg CdCl_2_·2.5 H_2_O; Cd+, 50 mg/kg CdCl_2_·2.5 H_2_O; Cd + NP1, 50 mg/kg Cd + 50 TiO_2_-NPs mg/L; Cd + NP2, 50 mg/kg Cd + 100 TiO_2_-NPs mg/L; Cd + NP3, 50 mg/kg Cd + 200 TiO_2_-NPs mg/L; Cd + NP4, 50 mg/kg Cd + 400 TiO_2_-NPs mg/L.

**Figure 2:**
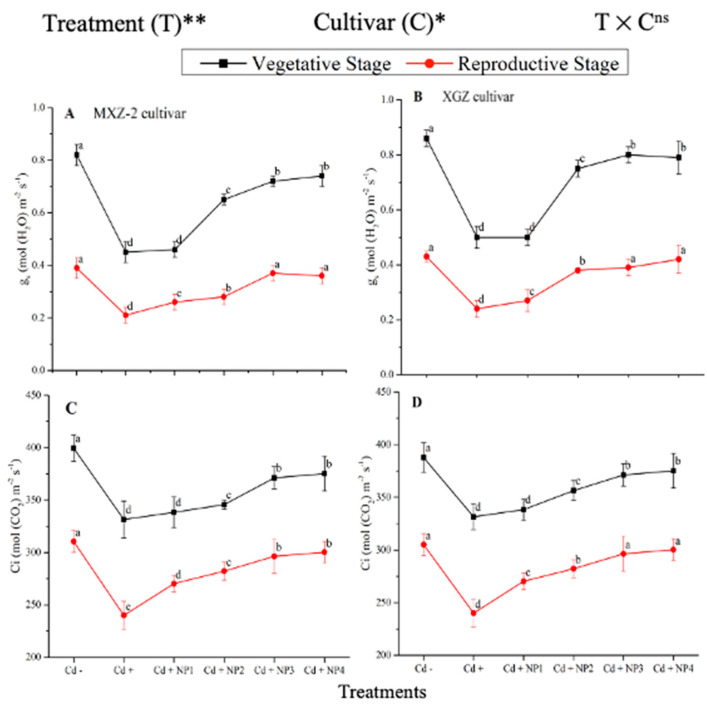
Effect of TiO_2_-NPs application and Cd stress on leaf stomatal conductance (**A**,**B**) and intercellular CO_2_ content (**C**,**D**) in the leaves of fragrant rice cultivars Meixiangzhan-2 (MXZ-2) and Xiangyaxiangzhan (XGZ) at the vegetative and reproductive stages. Tukey tests were used to compare means for the regimes in both stages, and a simple test based on the Tukey HSD test at 0.05 was used to interpret the results. Error bars are standard errors of the mean. Different letters, such as a, b, c & d above the lines, indicate statistical significance at *p* < 0.05. ^ns^ = non-significant; *, ** = significant at 5% and 1%, respectively. See Figure 1 for the treatment combination details.

Both fragrant rice cultivars, MXZ-2 and XGZ, showed significant differences (*p* < 0.05) in photosynthetic efficiency following TiO_2_-NP application during the vegetative and reproductive stages under Cd toxicity (Figure 1 and Figure 2). Similarly, leaf photosynthetic traits significantly differed among the cultivars under Cd toxicity. Under Cd stress, TiO_2_-NP application enhanced the leaf photosynthetic traits, such as *Pn*, Tr, g_s_, and Ci, compared with the Cd-only treatment (Cd+). Moreover, the treatments showed a similar trend at both growth stages. Averaged across the growth stages, Cd + TiO_2_-NP_3_ increased the leaf *Pn* and Tr by 62.6% and 39.1%, respectively, in MXZ-2 and by 69.4% and 44.6% in the XGZ cultivar, compared with control (Cd–) as shown in Figure 1. However, the high TiO_2_-NP (NP4) treated pots were statistically (*p* < 0.05) similar to NP3. Similarly, the low TiO_2_-NP (i.e., NP1 and NP2) treated pots also significantly enhanced leaf photosynthetic traits compared with Cd+-treated pots.

The differences in g_s_ and Ci were also significant among the different treatments at the vegetative and reproductive stages, significantly higher than the Cd-only treated pots (Figure 2A–D). Relative to exclusive Cd treatment; averaged across the growth stages; Cd + TiO_2_-NP3 increased the leaf g_s_ and Ci by 63.6% and 12.3%, respectively; in MXZ-2 and 59.4% and 16.8%, in XGZ cultivar; compared with Cd+. Likewise, the low TiO_2_-NP (i.e., NP1 and NP2) treated pots also significantly enhanced leaf g_s_ and Ci compared with Cd-only treatment. The results also suggested that compared to the MXZ-2 rice cultivar, XGZ demonstrated higher performance and was more responsive to TiO_2_-NPs.

### 3.2. Effect of TiO_2_-NPs on Leaf Stomatal Traits under Cd Toxicity

**Figure 3:**
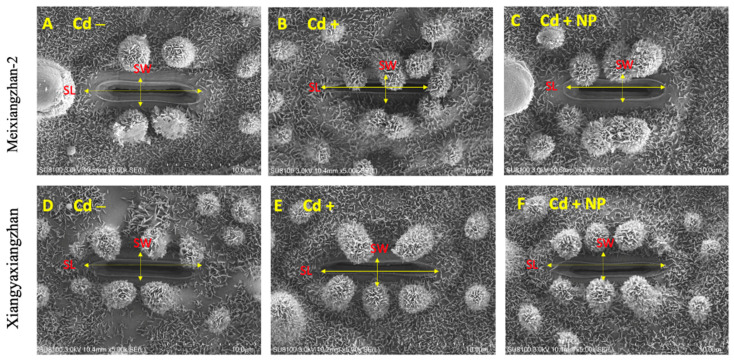
Effect of TiO_2_-NP application on fragrant rice cultivars, i.e., Meixiangzhan-2 and Xiangyaxiangzhan leaf stomatal length, width, and length compared with control (Cd−) and Cd (Cd+)-only treatments. Figures (**A**–**F**) represent the effect of treatments (Cd−, Cd+, and Cd + NP3) on leaf stomatal length and width. (**A**–**F**) ×5000 magnification, scale bars = 10 µm. SW: stomatal width, and SL: stomatal length. See Figure 1 for the treatment combination details.

**Figure 4:**
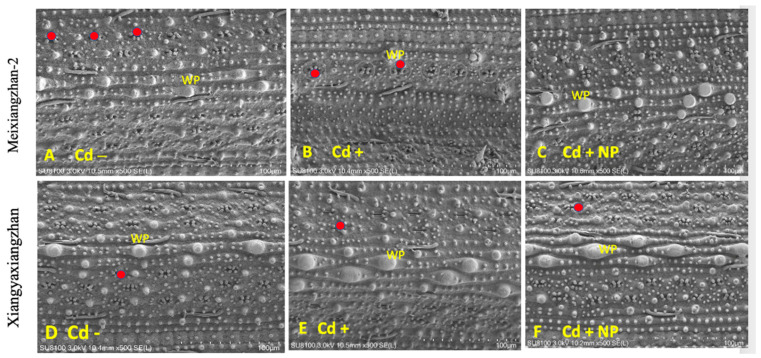
Effect of TiO_2_-NP application on leaf stomatal number of fragrant rice cultivars, i.e., Meixiangzhan-2 and Xiangyaxiangzhan. Figures (**A**–**F**) represent the effect of treatments (Cd−, Cd+, and Cd + NP3) on leaf stomatal number. (**A**–**F**) 10.5 mm × 500 SE magnification, scale bars = 100 µm. Red points show the stomata’s position. WP: wart-like protuberance. See Figure 1 for the treatment combination details.

Based on the study results observed for physiological and biochemical traits, it was found that the TiO_2_-NP3 treatment was more effective and significant than other regimes. Therefore, Cd−, Cd+, and Cd + TiO_2_-NP3 treatments were selected for leaf SEM analysis of both fragrant rice cultivars. The leaf SEM analysis revealed that Cd toxicity severely influenced the aromatic rice leaf stomatal traits, such as stomatal number, density, width, and length (Figure 3 and Figure 4). However, the TiO_2_-NP4-treated plants were observed to have a comparatively regular stomatal number length and width. The leaf SEM analysis showed that TiO_2_-NP4 treatment enhanced the stomatal length, density, and width by 23%, 32.4%, and 36.3%, respectively, in fragrant rice cultivar MXZ-2 and by 32.23%, 41.3%, and 45.34%, respectively, in XGZ.

### 3.3. Effect of TiO_2_-NPs on Antioxidant Enzyme Activity under Cd Toxicity

**Figure 5:**
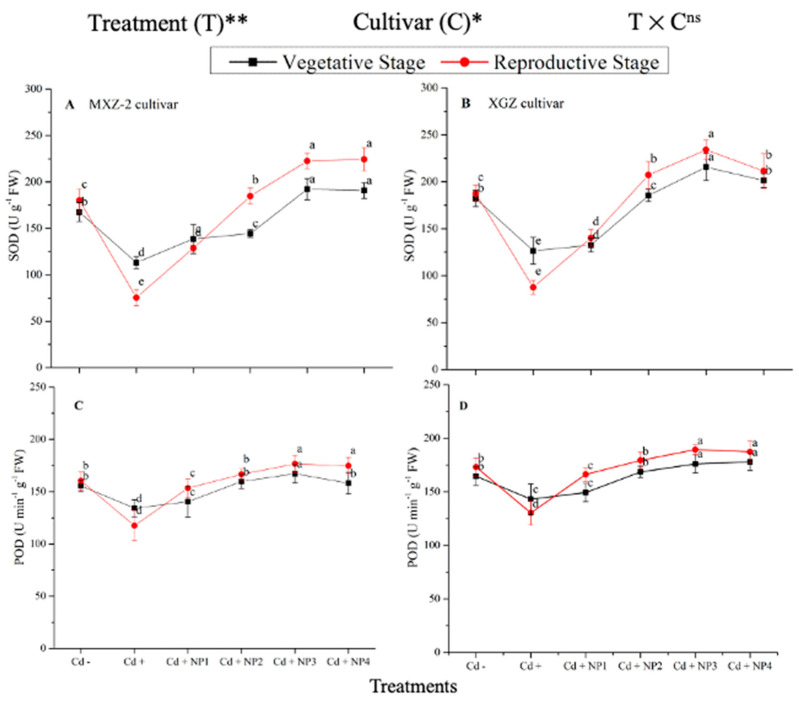
Effect of TiO_2_-NP application and Cd stress on the activity of superoxide dismutase (SOD) (**A**,**B**) and peroxidase (POD) (**C**,**D**) enzymes in the leaves of fragrant rice cultivars Meixiangzhan-2 (MXZ-2) and Xiangyaxiangzhan (XGZ) at the vegetative and reproductive stages. Tukey tests were used to compare means for the regimes in both stages, and a simple test based on the Tukey HSD test at 0.05 was used to interpret the results. Error bars are standard errors of the mean. Different letters, such as a, b, c, d & e above the lines, indicate statistical significance at *p* < 0.05. ^ns^ = non-significant; *, ** = significant at 5% and 1%, respectively. See Figure 1 for the treatment combinations.

**Figure 6:**
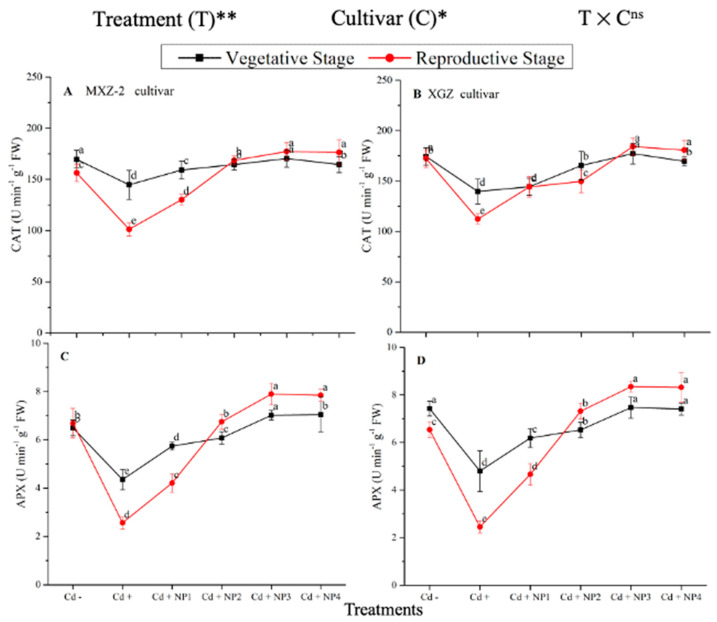
Effect of TiO_2_-NP application and Cd stress on catalase activity (**A**,**B**) and ascorbate peroxidase (APX) (**C**,**D**) enzymes in the leaves of fragrant rice cultivars Meixiangzhan-2 (MXZ-2) and Xiangyaxiangzhan (XGZ) at the vegetative and reproductive stages. Tukey tests were used to compare means for the regimes in both stages, and a simple test based on the Tukey HSD test at 0.05 was used to interpret the results. Error bars are standard errors of the mean. Different letters, such as a, b, c, d & e above the lines, indicate statistical significance at *p* < 0.05. ^ns^ = non-significant; *, ** = significant at 5% and 1%, respectively. See Figure 1 for the treatment combinations.

Several antioxidant enzyme activities were examined to investigate the function of TiO_2_-NP supplements to counteract Cd-induced oxidative stress in rice cultivars (Figure 5 and Figure 6). The results revealed that in both cultivars of fragrant rice, Cd stress considerably decreased the activity of the antioxidant enzymes compared to the Cd–treatment. Fragrant rice leaf antioxidant enzyme activities significantly differed among the cultivars, and a slighter decrease was noted in XGZ, showing that it is relatively more tolerant to Cd toxicity. Interestingly, TiO_2_-NP application alleviated the Cd toxicity effect under (Cd + TiO_2_-NP) treatments. Across the growth stages, the treatments showed a similar trend. Averaged across the growth stages, Cd + TiO_2_-NP3 treatment significantly enhanced the SOD (120.5 and 110.4%), POD (36.6 and 34.2%), CAT (41.4 and 43.8%), and APX (116.2 and 123.4%) enzyme activities in MXZ-2 and XGZ fragrant rice, respectively, as compared to Cd-only (Cd+) treatment (Figure 5 and Figure 6). However, the high NP (NP4)-treated pots were statistically (*p* < 0.05) similar to NP3. Likewise, the low NP (i.e., NP2 and NP3) pots also considerably enhanced antioxidant enzyme activities compared with Cd-only treated pots.

### 3.4. Effect of TiO_2_-NPs on Antioxidant Enzyme Transcript Levels under Cd Toxicity

**Figure 7:**
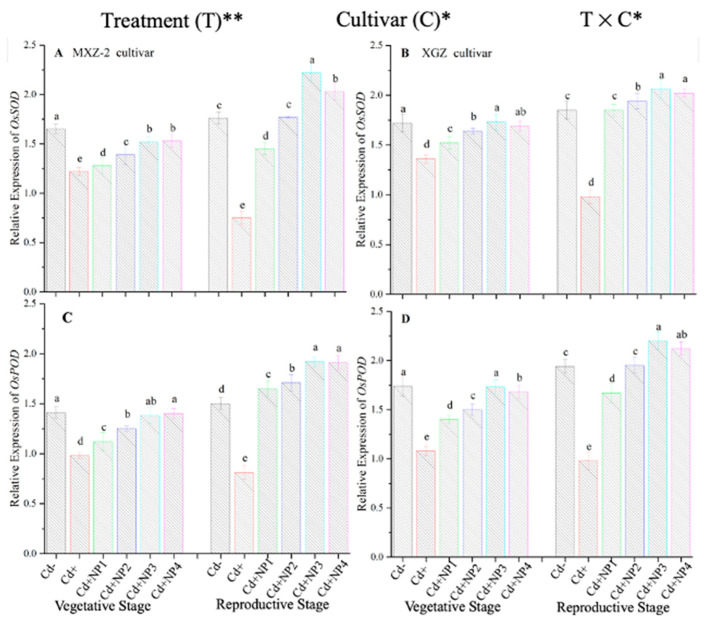
Effect of TiO_2_-NP application on the expression *OsSOD* (**A**,**B**) and *OsPOD* (**C**,**D**) enzymes in the leaves of fragrant rice cultivars Meixiangzhan-2 (MXZ-2) and Xiangyaxiangzhan (XGZ) at the vegetative and reproductive stages under Cd stress. Tukey tests were used to compare means for the regimes in both stages, and a simple test based on the Tukey HSD test at 0.05 was used to interpret the results. Error bars are standard errors of the mean. Different letters, such as a, ab, b, c, d & e above the columns, indicate statistical significance at *p* < 0.05. *, ** = significant at 5% and 1%, respectively. See Figure 1 for the treatment combinations.

**Figure 8:**
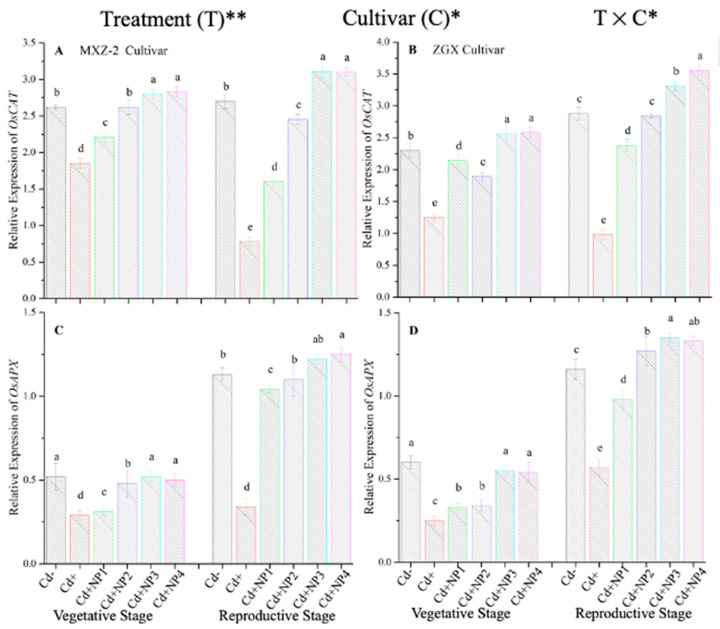
Effect of TiO_2_-NP application and Cd stress on the expression *OsCAT* (**A**,**B**) and *OsAPX* (**C**,**D**) enzymes in the leaves of fragrant rice cultivars Meixiangzhan-2 (MXZ-2) and Xiangyaxiangzhan (XGZ) at the vegetative and reproductive stages. Tukey tests were used to compare means for the regimes in both stages, and a simple test based on the Tukey HSD test at 0.05 was used to interpret the results. Error bars are standard errors of the mean. Different letters, such as a, ab, b, c, d & e above the columns, indicate statistical significance at *p* < 0.05. *, ** = significant at 5% and 1%, respectively. See Figure 1 for the treatment combinations.

The expression pattern of antioxidant encoding genes in fragrant rice cultivars is shown in Figure 7 and Figure 8. In this work, the expression levels of antioxidant-encoding genes were altered under treatments. Related to Cd–, the expression levels of antioxidant-encoding genes (i.e., *OsSOD, OsPOD, OsCAT*, and *OsAPX*) in both rice cultivars were significantly lower under Cd toxicity. However, TiO_2_-NP application increased the expression pattern of *OsSOD, OsPOD, OsCAT*, and *OsAPX* genes under Cd toxicity. Relative to Cd+, averaged across the growth stages, Cd + TiO_2_-NP3 significantly increased the transcript levels of *OsSOD* (90.8 and 66.6%), *OsPOD* (85.3 and 90.2%), *OsCAT* (125.1 and 163.8%), and *OsAPX* (171.3 and 65.7%) in fragrant rice cultivars MXZ-2 and XGZ, respectively (Figure 7 and Figure 8). Similarly, the other combined treatments (Cd + TiO_2_-NPs) also considerably enhanced the transcript levels of antioxidant-encoding genes.

### 3.5. Effect of TiO_2_-NPs on Proline and Soluble Protein Contents under Cd Stress

**Figure 9:**
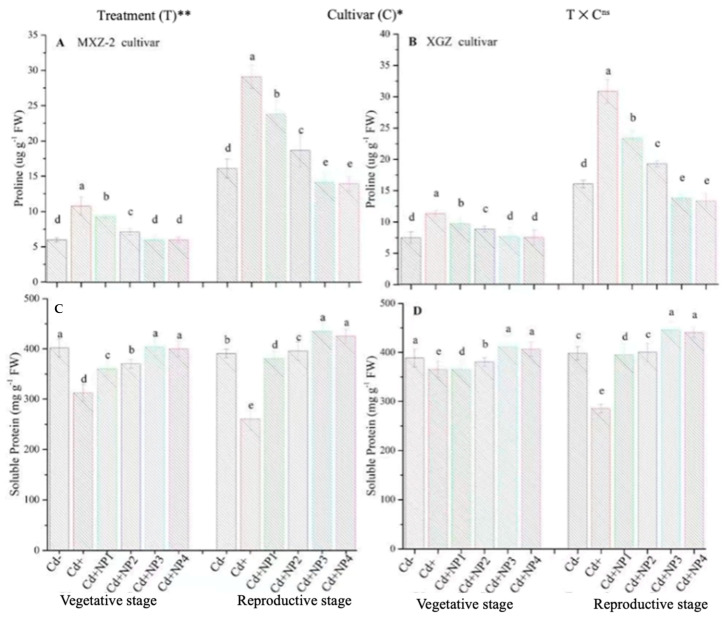
Effect of TiO_2_-NP application and Cd stress on the proline content (**A**,**B**) and soluble protein (**C**,**D**) enzymes in the leaves of fragrant rice cultivars Meixiangzhan-2 (MXZ-2) and Xiangyaxiangzhan (XGZ) at the vegetative and reproductive stages. Tukey tests were used for to compare means for the regimes in both stages, and a simple test based on the Tukey HSD test at 0.05 was used to interpret the results. Error bars are standard errors of the mean. Different letters, such as a, b, c, d & e above the columns, indicate statistical significance at *p* < 0.05. ^ns^ = non-significant; *, ** = significant at 5% and 1%, respectively. See Figure 1 for the treatment combinations.

Proline and soluble protein contents were considerably altered under the combined application of Cd and TiO_2_-NPs in both cultivars (Figure 9). Likewise, significant differences were noted in both cultivars for protein and proline content under Cd toxicity. Proline content was considerably increased under Cd toxicity at both stages compared to non-Cd treated pots (Figure 9A,B). However, averaged across the growth stages, TiO_2_-NP4 application decreased leaf proline content by 57.4% and 64.3% in MXZ-2 and XGZ cultivars, respectively, relative to Cd+. Similarly, the lower treatments of NPs also decreased proline content significantly compared with the Cd-only treatment. In contrast to proline, the Cd-only pots considerably decreased the leaf soluble protein contents. Linear increments in soluble protein contents were noted from the vegetative to reproductive growth stages. Relative to Cd+, Cd + TiO_2_-NP3-treated pots significantly enhanced the leaf soluble protein content by 43.6% and 32.2% in MXZ-2 and XGZ cultivars (Figure 9C,D). Furthermore, the results showed that the leaf proline content and soluble protein in MXZ-2 was lower than in XGZ, indicating that of the two cultivars, MXZ-2 is more susceptible to Cd stress than XGZ.

### 3.6. Effect of TiO_2_-NPs on Fragrant Rice Leaf MDA and H_2_O_2_ Content under Cd Stress

**Figure 10:**
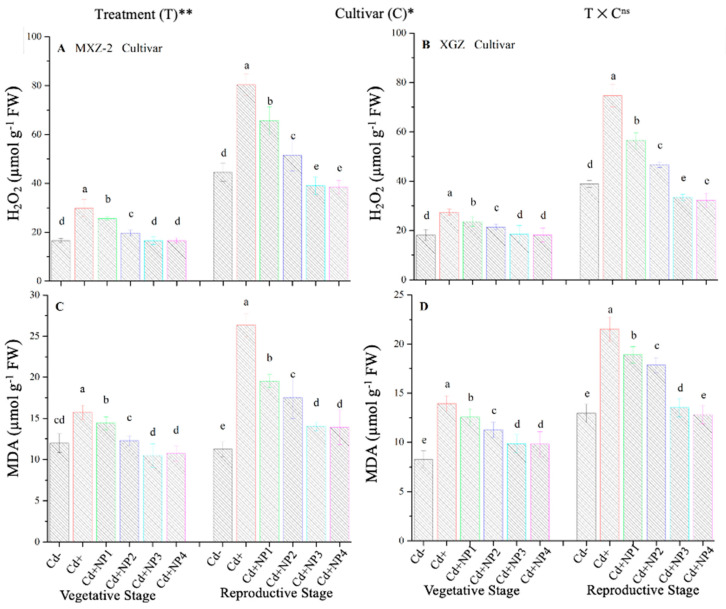
Effect of TiO_2_-NP application on hydrogen peroxide (H_2_O_2_) (**A,B**) and malondialdehyde (MDA) (**C**,**D**) enzymes in the leaves of fragrant rice cultivars MXZ-2 and XGZ at the vegetative and reproductive stages under Cd stress. Tukey tests were used to compare means for the regimes in both stages, and a simple test based on the Tukey HSD test at 0.05 was used to interpret the results. Error bars are standard errors of the mean. Different letters, such as a, b, c, d & e above the columns, indicate statistical significance at *p* < 0.05. ^ns^ = non-significant; *, ** = significant at 5% and 1%, respectively. See Figure 1 for the treatment combinations.

The contents of MDA and H_2_O_2_ were found to be altered under different treatments in this study (Figure 10). Results revealed that TiO_2_-NP application significantly decreased the concentration of MDA and H_2_O_2_ in leaves of both fragrant rice cultivars under Cd toxicity. Similarly, significant (*p* < 0.05) differences were observed between XGZ and MXZ-2 for leaf MDA and H_2_O_2_ content under Cd toxicity. In contrast, Cd-only pots significantly increased the concentration of MDA and H_2_O_2_ in the leaves of both rice cultivars compared with Cd– and Cd+ TiO_2_-NP treatment pots. Compared with soil Cd pots (Cd–), Cd-stressed pots significantly increased the content of MDA by 84.7 and 67.4% and H_2_O_2_ by 80.3% and 74.6%, respectively, in MXZ-2 and XGZ cultivars (Figure 10A–D). Moreover, the results revealed that the concentration of H_2_O_2_ and MDA in MXZ-2 was higher than in XGZ, indicating that fragrant rice cultivar XGZ is relatively more tolerant to Cd toxicity than MXZ-2.

### 3.7. Effect of TiO_2_-NPs on Cd Uptake and Accumulation in Fragrant Rice Parts

Cd accumulation in roots, stems, leaves, and grains were higher under Cd-stress conditions (Table 1). The application of TiO_2_-NPs resulted in a significant decrease in roots, leaves + stem, and grain Cd contents under Cd toxicity treatment. Moreover, both aromatic rice cultivars, XGZ and MXZ-2, were also considerably different for the measured parameters. The Cd accumulation revealed the following pattern: root > stem + leaves > grains. The outcomes showed that the higher dose of TiO_2_-NP application significantly decreased the concentration and accumulation of Cd in different parts (i.e., roots, stems, leaves, and grains) of both fragrant rice cultivars under Cd toxicity. Furthermore, the results showed that the contents and accumulation of Cd in MXZ-2 were higher than in XGZ, indicating that XGZ is relatively more tolerant to Cd stress than the fragrant rice cultivar MXZ-2.

### 3.8. Relationship between Net Photosynthetic Rate, Leaf Proline, and Soluble Protein Content

**Figure 11:**
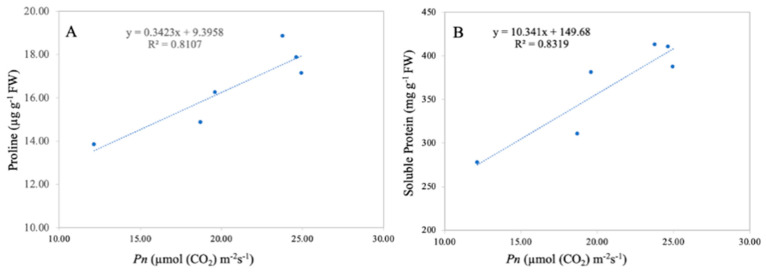
Linear relationship among leaf net photosynthetic rate and leaf proline (**A**) and protein content (**B**) under combined TiO_2_-NP application and Cd. *n* = 6.

A significant correlation between the leaf net photosynthetic rate and leaf proline and soluble protein content was observed in the current study (Figure 11). Linear regression analysis revealed that the leaf net photosynthetic rate was positively correlated with leaf proline content (R^2^ = 0.81 **) and with the leaf soluble protein content (R^2^ = 0.83 **). The correlation analysis revealed that the leaf net photosynthetic rate is directly related to leaf proline and soluble proline content, suggesting that a higher net photosynthetic rate results in higher leaf proline and soluble proline content.

## 4. Discussion

The heavy metal Cd is a non-essential and toxic trace element for crops. Cd accumulation in plants can severely inhibit growth, antioxidant systems, yield, and quality of crops by influencing plant physiological, biochemical, and molecular processes [35,45]. Therefore, Cd-stress-allaying strategies in crop growth and development, grain yield reduction, and quality deterioration through strengthened plant metabolism remain hot topics for plant researchers. TiO_2_-NPs have been used as nano fertilizers to increase crop growth and yield and reduce metal accumulation in plants because of their primary role in crop growth and production and in increasing plant oxidative stress resistance under heavy metal stress [25,46]. We investigated two different fragrant rice cultivars that responded differently to Cd stress. The main purpose of our study was to explore how TiO_2_-NPs could alleviate the negative effect of Cd toxicity in Cd-sensitive and Cd-resistant fragrant rice cultivars. To our knowledge, this is the first study to investigate the effect of TiO_2_-NPs on the physio-biochemical process of fragrant rice. The present research investigated various alleviating roles of TiO_2_-NP supply on leaf photosynthetic traits, antioxidant defense system, and biochemical characteristics of two rice cultivars under Cd stress.

Assessing leaf photosynthetic efficiency is a cost-efficient and quick technique that might help explore plant fitness, particularly under stress conditions [47]. In the current study, our results demonstrated that Cd stress considerably decreased the leaf photosynthetic activity at the vegetative and reproductive growth stages of both fragrant rice cultivars, i.e., MXZ-2 and XGZ (Figure 1 and Figure 2), which are in line with the findings of previous studies, which reported the Cd-induced decrease in leaf photosynthetic efficiency [45,48]. A possible explanation for the decrease in leaf photosynthetic activity may be that a higher accumulation of Cd can inhibit the uptake of all essential nutrients via plant roots [49,50,51]. Moreover, SEM analysis of the leaf in this study showed that the leaf stomatal traits, such as stomatal number, width, and length, were highly affected by Cd stress (Figure 3 and Figure 4). Relative to the Cd– treatment, the Cd-stressed plants significantly decreased their stomatal number, density, width, and length in this study.

In the current study, we discovered that the application of TiO_2_-NPs significantly increased the leaf photosynthetic attributes in fragrant rice plants under Cd stress (Figure 1 and Figure 2), which may be attributed to TiO_2_-NP-induced reductions in oxidative damage, leaf chloroplast ultra-structure, and leaf stomatal traits (Figure 3). Similarly, earlier researchers reported that TiO_2_-NP application enhanced photosynthetic leaf pigments, photosynthetic activity, and chloroplast integrity in rice [29,46]. A possible explanation for the improvements in leaf photosynthetic efficiency might be associated with increments in leaf stomatal traits in TiO_2_-NP-treated plants. The finding of this study was also in line with the outcomes reported by Sing & Lee [51].

Antioxidants play a crucial role in plant defense mechanisms and can reduce reactive oxidative stress and oxidative damage in plants [52]. In the current study, our outcomes revealed that antioxidant enzyme activity was disturbed by the availability of Cd in the soil, while TiO_2_-NP application improved the antioxidant enzyme activity in fragrant rice under Cd toxicity (Figure 5 and Figure 6). Moreover, the current study revealed that Cd stress causes oxidative damage in rice leaves. At the same time, TiO_2_-NP application healed oxidative damage, which could be associated with the increment of the antioxidant enzyme activity and antioxidant-encoding gene expression level (Figure 5, Figure 6, Figure 7 and Figure 8). Previous studies reported that the safety of plants by antioxidant enzyme activities against oxidative plant damage was countered by SOD, POD, CAT, and APX [53,54]. In this study, our results further revealed reduced SOD activity in Cd-treated pots. This may be because SOD is the first line of defense in the antioxidant system, controlling reactive oxidative stress and converting toxic O_2_ to less toxic H_2_O_2_ [55,56]. A similar pattern was noted in CAT and other antioxidant enzyme activity; basically, CAT helps to avert oxidative damage in plant cells by changing O_2_ to less toxic H_2_O_2_ [57]. The profound decline in antioxidant enzyme activity under Cd stress might be due to the process involved in removing O_2_ and/or higher production of MDA and H_2_O_2_ in the current study (Figure 10). However, TiO_2_-NPs application improved antioxidant enzyme activity across the growth stages of both fragrant rice cultivars, which might play a key role in decreasing plant damage in the current study. Moreover, the expression levels of antioxidant encoding genes, such as *OsSOD, OsPOD, OsCAT*, and *OsAPX*, in both fragrant rice cultivars, were highly expressed in TiO_2_-NP-treated plants compared to Cd+-treated plants. Gao et al. [29] reported that nano-TiO_2_ promotes the growth and development of plants by protecting the chloroplast sheath assembly from the reactive O_2_, consequently increasing the function of the antioxidant enzymes.

Proline is an osmotic controlling component found in plant cytoplasm that helps to maintain cell osmotic pressure by influencing cell water potential [58]. In the current study, leaf proline content significantly increased under the Cd-stress conditions (Figure 9). Heavy metal stress, particularly Cd toxicity, increases plant proline levels due to resistance of plants under stress [59]. The enhancement in plant proline content can be associated with plant protein degradation [60]. An enhancement in plant tissue proline levels can reflect plant injury [60]. The TiO_2_-NP application improves leaf defensive systems and decreases the leaf proline content at different growth stages in the current study, suggesting the ameliorating role in maintaining plant osmotic balance under Cd toxicity (Figure 9A,B). Moreover, the correlation analysis revealed that the leaf’s net photosynthetic rate strongly correlates with leaf proline content (Figure 11).

Figure 9C,D show the effects of TiO_2_-NP application on the fragrant rice cultivars’ leaf protein contents under Cd stress. In this study, the Cd toxicity decreased the protein content in rice leaves, which might be due to the greater oxidative damage. Our results align with earlier work showing that Cd stress stimulated protein deprivation through more significant protease activity [45,61]. Similarly, the harmful effect of Cd on soluble protein has also been reported in other studies [60,61], However, the TiO_2_-NP application countered the adverse effect of Cd toxicity and significantly improved the protein content in fragrant rice leaves in the current work. Similarly, in earlier studies, an increase in protein content with the application of TiO_2_-NPs has been observed for barley [62] and wheat [63]. These outcomes show that TiO_2_-NPs increased the yield of the plant-soluble protein content of various crops by enhancing plant essential nutrient uptake and accumulation. In addition, the linear regression analysis also showed that the leaf net photosynthetic rate is highly and significantly correlated with soluble proline content, suggesting that a higher net photosynthetic rate results in a higher leaf soluble proline content (Figure 11).

Lipid peroxidation indicates the presence of free radical association in plant tissues and is typically expressed as the MDA level, an important indicator of lipid peroxidation caused by oxidative plant damage [58]. The present experiment showed that Cd stress stimulated the plant’s oxidative damage, as evident by greater production of MDA and H_2_O_2_ (Figure 10). However, TiO_2_-NP application significantly decreased the MDA and H_2_O_2_ in fragrant rice leaf tissues at vegetative and reproductive growth stages, suggesting that TiO_2_-NP application alleviated Cd-triggered intracellular membrane disruptions during growth.

Cd is a non-essential metal and is toxic to human health via the terrestrial food chain [64]. Therefore, Cd-contaminated rice has become a severe problem in many countries. In the current study, TiO_2_-NP application significantly decreased the Cd content and accumulation in both fragrant rice cultivars under Cd stress (Table 1). The Cd + NP3 treatment significantly decreased the Cd content in different plant parts, such as roots, leaves + stem, and grains, compared to the Cd-only pots. A possible explanation for the decreasing content of Cd in rice plants is that TiO_2_-NPs regulated the rate of transpiration in leaves, thereby modulating the transport of Cd within the plant as Cd translocation follows water transport upwards in the xylem [65]. Previous studies have reported that exposure of plants to co-application of Cd and TiO_2_-NPs via soil decreased Cd uptake and translocation [66,67]. This report apparently agreed with the results obtained in our study on tissue Cd accumulation. As shown in Table 1, co-exposure treatments reduced the Cd contents in plant tissues (roots, leaves, and seeds) compared to the Cd-only treatment. This was consistent with the results of Ji et al. [66] in rice seedlings, although the route of exposure of TiO_2_-NPs was the soil medium. The presence of TiO_2_-NPs in plant tissues could have initiated the production of extracellular polypeptides such as phytochelatins (not studied here), which can immobilize Cd in tissues and reduce translocation. The results indicate that the soil application of TiO_2_-NPs is an effective strategy to reduce the transfer of Cd into the food chain from moderately contaminated soils.

## 5. Conclusions

In the current study, we investigated two different fragrant rice cultivars that responded differently to Cd stress. To our knowledge, this is the first study to investigate the effect of TiO_2_-NPs on the physio-biochemical process of fragrant rice. The main purpose of our study was to explore how TiO_2_-NPs could alleviate the negative effect of Cd toxicity in Cd-sensitive and Cd-resistant fragrant rice cultivars. The results showed that Cd toxicity affected plant growth, leaf physiology, and metabolic activity. Cd stress enhanced the production of proline, MDA, and H_2_O_2_ in fragrant rice leaves, apparently by desynchronizing the reactive oxidative stress scavenging mechanism. Moreover, Cd uptake and accumulation in roots, stems, leaves, and grains were higher under Cd-stress conditions. However, TiO_2_-NP application competently counteracted the adverse effects of Cd toxicity on the physiological and biochemical parameters related to plant growth and development, which could primarily be attributed to decreased Cd accumulation and improved plant physiological and antioxidant systems. Therefore, our outcomes concluded that TiO_2_-NP application alleviated the Cd-provoked inhibitory influences on leaf photosynthetic traits, antioxidant enzyme activity, and protein degradation throughout the growth period, thereby improving the plant’s physiological activity and defense system.

## Figures and Tables

**Table 1:** Effect of TiO_2_-NP application on Cd accumulation in fragrant rice cultivars under Cd stress.

Cultivar	Treatments	Cd Content (µg g^−1^ DW)		Grain
		Root	Stem + Leaf	
	Cd–	13.76 ± 1.78 ^e^	3.43 ± 0.45 ^f^	0.13 ± 0.02 ^e^
	Cd+	216.76 ± 12.43 ^a^	46.87 ± 3.43 ^a^	1.45 ± 0.08 ^a^
	Cd + NP1	140.98 ± 9.34 ^b^	23.87 ± 2.32 ^b^	0.87 ± 0.04 ^b^
Meixiangzhan-2	Cd + NP2	118.44 ± 10.30 ^b^	16.98 ± 1.88 ^c^	0.45 ± 0.03 ^c^
	Cd + NP3	71.98 ± 4.46 ^c^	12.98 ± 1.12 ^d^	0.22 ± 0.01 ^d^
	Cd+ NP4	26.87 ± 2.32 ^d^	7.87 ± 0.87 ^e^	0.21 ± 0.01 ^d^
Average		98.13 ^a^	18.66 ^a^	0.55 ^a^
	Cd –	11.53 ± 2.02 ^e^	2.51± 0.60 ^f^	0.08 ± 0.01 ^d^
	Cd+	196.23 ± 11.30 ^a^	31.23 ± 3.25 ^a^	1.05 ± 0.10 ^a^
Xiangyaxiangzhan	Cd + NP1	123.34 ± 12.43 ^b^	16.33 ± 2.10 ^b^	0.87± 0.04 ^b^
	Cd + NP2	105.43 ± 7.34 ^b^	12.12 ± 1.24 ^c^	0.55 ± 0.05 ^c^
	Cd + NP3	55.12 ± 6.64 ^c^	9.24 ± 1.85 ^d^	0.11 ± 0.02 ^d^
	Cd + NP4	24.12 ± 2.12 ^d^	8.34 ± 0.98 ^e^	0.10 ± 0.01 ^d^
Average		85.94 ^b^	13.29 ^b^	0.46 ^b^
ANOVA				
Treatments (T)		**	**	**
Cultivars (C)		*	*	*
T × C		ns	ns	ns

Values are the means of three replicates ± SE. Values with different letters (such as a, b, c, d, e and f) differ significantly at *p* < 0.05 based on the Tukey HSD test. ^ns^ = non-significant; *, ** = significant at 5% and 1%, respectively; Cd–, 0 mg/kg CdCl_2_·2.5 H_2_O; Cd+, 50 mg/kg CdCl_2_·2.5 H_2_O; Cd + NP1, 50 mg/kg Cd + 50 TiO_2_-NPs mg/L; Cd + NP2, 50 mg/kg Cd + 100 TiO_2_-NPs mg/L; Cd + NP3, 50 mg/kg Cd + 200 TiO_2_-NPs mg/L; Cd + NP4, 50 mg/kg Cd + 400 TiO_2_-NPs mg/L.

## Data Availability

The data presented in this study are available on request from the corresponding author. The data are not publicly available due to privacy.

## Supplementary Materials

The following supporting information can be downloaded at: https://www.mdpi.com/article/10.3390/metabo13060765/s1, Table S1: Soil chemical properties of the experimental soil prior to experimentation; Table S2: Primer sequences used for qRT-PCR amplification.
