# Supplementary material for: Exogenous TiO2 Nanoparticles Alleviate Cd Toxicity by Reducing Cd Uptake and Regulating Plant Physiological Activity and Antioxidant Defense Systems in Rice (Oryza sativa L.)
Source: Metabolites. 2023 Jun 19;13(6):765. doi: 10.3390/metabo13060765 (PMC10303181; doi:10.3390/metabo13060765)
Supplement: Supplementary file 1 [file metabolites-13-00765-s001.zip › metabolites-2372533-supplementary.pdf]

**Table S1:** Soil chemical properties of the experimental soil prior to experimentation.

| Properties | Contents |
|---|---|
| Organic matter (g $kg^{-1}$) | 20.78 |
| Total nitrogen (g $kg^{-1}$) | 1.16 |
| Total phosphorus (g $kg^{-1}$) | 0.99 |
| Total potassium (g $kg^{-1}$) | 18.85 |
| pH | 5.87 |
| Available nitrogen (mg $kg^{-1}$) | 90.45 |
| Available phosphorus (mg $kg^{-1}$) | 9.49 |
| Available potassium (mg $kg^{-1}$) | 127.91 |
| Available molybdenum (mg $kg^{-1}$) | 0.081 |
| Cadmium (mg $kg^{-1}$) | 4.26 |

**Table S2:** Primer sequences used for qRT-PCR amplification

| Genes | Strand | 5′ to 3′ Primer Sequences | Annealing Temperature | Accession no. |
|---|---|---|---|---|
| OsSOD | Forward | TGTCAACTGGACCACACTTC | 58 °C | Os07g0665200 |
| | Reverse | ACTTAAAACGCATGCACTCA | | |
| OsPOD | Forward | CGACGATTTCTACGACTACAT | 59 °C | Os10g0109600 |
| | Reverse | TGATTGAGGAGGTTCTGGT | | |
| OsCAT | Forward | GCACAGTTTGACAGGGAG | 55 °C | Os06g51150 |
| | Reverse | GTCTTTGGACTTGGCTTG | | |
| OsAPX | Forward | TACGCCGACTTCTACCAGC | 57 °C | Os07g0694700 |
| | Reverse | TTTATTACAACCGCCACGA | | |
| ACTIN | Forward | TGCCAAGGCTGAGTACGACGA | 58 °C | Os03g50885 |
| | Reverse | CAAGCAGGAGGACGGCGATA | | |
